# Manganese‐Doping‐Induced Quantum Confinement within Host Perovskite Nanocrystals through Ruddlesden–Popper Defects

**DOI:** 10.1002/anie.201914473

**Published:** 2020-03-05

**Authors:** Sharmistha Paul, Eva Bladt, Alexander F. Richter, Markus Döblinger, Yu Tong, He Huang, Amrita Dey, Sara Bals, Tushar Debnath, Lakshminarayana Polavarapu, Jochen Feldmann

**Affiliations:** ^1^ Chair for Photonics and Optoelectronics Nano-Institute Munich Department of Physics Ludwig-Maximilians-Universität (LMU) Königinstrasse 10 80539 Munich Germany; ^2^ EMAT University of Antwerp Groenenborgerlaan 171 2020 Antwerp Belgium; ^3^ Department of Chemistry Ludwig-Maximilians-Universität München Butenandtstrasse 5–13 (E) 81377 Munich Germany

**Keywords:** CsPbX_3_ nanocrystals, exciton properties, manganese-doped perovskite nanocrystals, quantum confinement, Ruddlesden–Popper defects

## Abstract

The concept of doping Mn^2+^ ions into II–VI semiconductor nanocrystals (NCs) was recently extended to perovskite NCs. To date, most studies on Mn^2+^ doped NCs focus on enhancing the emission related to the Mn^2+^ dopant via an energy transfer mechanism. Herein, we found that the doping of Mn^2+^ ions into CsPbCl_3_ NCs not only results in a Mn^2+^‐related orange emission, but also strongly influences the excitonic properties of the host NCs. We observe for the first time that Mn^2+^ doping leads to the formation of Ruddlesden–Popper (R.P.) defects and thus induces quantum confinement within the host NCs. We find that a slight doping with Mn^2+^ ions improves the size distribution of the NCs, which results in a prominent excitonic peak. However, with increasing the Mn^2+^ concentration, the number of R.P. planes increases leading to smaller single‐crystal domains. The thus enhanced confinement and crystal inhomogeneity cause a gradual blue shift and broadening of the excitonic transition, respectively.

## Graphical abstract

**The doping** of Mn^2+^ ions into CsPbCl_3_ nanocrystals (NCs) strongly influences the excitonic properties of the host NCs. Manganese doping leads to the formation of Ruddlesden–Popper defects and thus induces quantum confinement within individual crystals. This results in a blue shift of the excitonic emission.

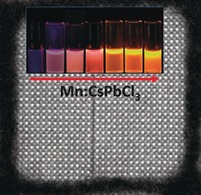

Halide perovskite nanocrystals (NCs) became popular in recent years not only because of their extraordinary optical and optoelectronic properties, but also owing to their technological applications.^1^ The optical properties of perovskite NCs are generally tunable through their halide composition and morphology. In addition, they can also be tuned by doping with metal‐ion‐based luminescent activators. The past two decades have witnessed a great progress in the field of doped colloidal semiconductor NCs.^2^ In such NCs, the excitons of host NCs strongly interact with the dopants, thus eventually leading to dopant emission.2a, 2b In particular, Mn^2+^‐doping of II–VI semiconductor NC hosts is the most investigated case, which already resulted in an understanding of exciton‐to‐dopant energy and electron‐transfer mechanisms.2a, 3 The transfer of the exciton energy from a semiconductor host to Mn^2+^ dopants leads to orange emission from a spin‐forbidden ^4^T_1_–^6^A_1_ Mn d–d transition.3a, 4 Recently, this concept has been extended to halide perovskite NCs.^5^ Over the last two years, intense research efforts have been devoted to the synthesis and optical investigation of Mn^2+^ doped halide perovskite NCs of different morphologies and halide compositions.^5^, ^6^ Among all, CsPbCl_3_ NCs are the best host matrix for efficient energy transfer from the exciton to the Mn^2+^ dopants.

To date, most of the research on Mn^2+^ doped NCs has mainly focused on the enhancement of the dopant emission via energy transfer mechanisms and thermal stability.6b, 6i, 6j, 7 It has been widely reported that at higher Mn concentration, strong Mn^2+^–Mn^2+^ interactions can be observed. This leads to a drastic reduction of the Mn^2+^‐related photoluminescence (PL) yield because of the formation of Mn^2+^ doping induced defects in the mid band‐gap of the host NCs. The formation of such small Mn^2+^ clusters can enhance electron trapping leading to non‐radiative recombination. Therefore, it is of utmost important to address the Mn^2+^ doping induced lattice arrangements and the subsequent effect on the exciton properties. However, the effect of Mn^2+^ doping on the excitonic properties of perovskite NCs is still largely unexplored. Moreover, the influence of the Mn^2+^ dopants on the host perovskite crystal lattice is unclear, especially because there is significant variation in the sizes of Mn^2+^ (1.6 Å) and Pb^2+^ (2.38 Å). Previous studies claimed that the Mn replaces Pb in perovskite crystal lattice6c and uniformly distributes throughout host matrix.6k Herein, we find that the doping of Mn^2+^ ions into CsPbCl_3_ NCs strongly influences the excitonic properties of host perovskite NCs. The excitonic absorption peak becomes prominent and the PL quantum yield (QY) of the excitonic emission enhances at low dopant concentrations. However, at high doping, quantum confinement is induced in the host NCs through Ruddlesden‐Popper (R.P.) defects, as revealed by atomically resolved high‐angle annular dark‐field scanning transmission electron microscopy (HAADF‐STEM).

Colloidal Mn^2+^‐doped CsPbCl_3_ NCs were prepared by slightly modifying the ligand‐assisted ultrasonication approach for perovskite NCs, which was previously developed by our group.^8^ A schematic illustration of the synthesis process and the precursors used in the synthesis is shown in Figure 1 a. Briefly, 10 mL of 1‐octadecene (ODE) solvent and ligand solutions (1 mL of oleylamine and 1 mL of oleic acid) were added to a mixture of precursor powders (CsOAc, PbCl_2_, and MnCl_2_) in a 20 mL glass bottle. The reaction mixture was subjected to tip‐sonication for 15 minutes under ambient conditions (see experimental section in the Supporting Information for more details). The reaction mixture gradually turns to white during ultrasonication, indicating the formation of perovskite NCs. The colloidal dispersion obtained exhibits orange emission under UV light illumination, indicating the successful doping of Mn^2+^ ions into CsPbCl_3_ NCs. This reaction is scalable and large quantities of bright luminescent Mn^2+^‐doped NCs can be obtained in powder form (Figure S1). For comparison, we also prepared pure CsPbCl_3_ NCs under similar experimental conditions. As depicted in Figure 1 c, the colloidal dispersion of CsPbCl_3_ NCs exhibits an excitonic absorption peak at 390 nm and a PL peak at 403 nm, which are typical for bulk‐like CsPbCl_3_ NCs. On the other hand, the PL spectrum of colloidal NCs obtained in the presence of MnCl_2_ (MnCl_2_:PbCl_2_=1.7:1) in the reaction medium exhibits an additional PL peak at 600 nm corresponding to the Mn^2+^ d–d transition, indicating the successful incorporation of Mn^2+^ ions into host CsPbCl_3_ NCs. Interestingly, the first excitonic absorption peak appears to be more prominent for Mn^2+^‐doped CsPbCl_3_ NCs as compared to undoped NCs (Figures 1 c,d). Moreover, the peak at higher energy is also more pronounced for doped NCs. In addition, we find that the PLQY of the excitonic emission increases from around 0.5 % to around 3 % (the PLQY of CsPbCl_3_ NCs is generally low) after Mn^2+^ doping, despite the energy transfer from excitons to Mn^2+^ dopants. The PLQY should be even higher if there is no energy transfer to dopants that leads to strong Mn‐related PL at 600 nm. These results clearly suggest that Mn^2+^ doping enhances the excitonic transitions of the host CsPbCl_3_ perovskite NCs.

**Figure 1:**
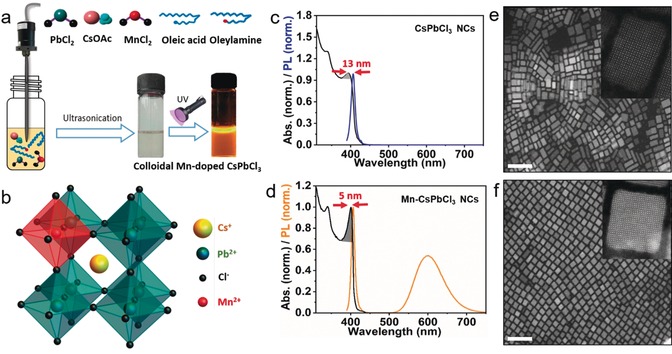
a) Schematic illustration of the synthesis of Mn^2+^‐doped CsPbCl_3_ NCs by ultrasonication and the photograph of the obtained colloidal solution under room light and UV light illumination. b) Atomic model of Mn^2+^‐doped CsPbCl_3_ perovskite crystal structure. c),d) Normalized UV/Vis absorption and PL spectra of undoped and Mn^2+^‐doped CsPbCl_3_ perovskite NCs, respectively, and corresponding HAADF‐STEM images (e,f), scale bar: 50 nm. Insets in (e,f) are corresponding high‐resolution HAADF‐STEM images.

Undoped CsPbCl_3_ NCs generally exhibit a very low PLQY, owing to vacancies in their crystal lattice and surface defects. It is well established that defects and trapped charges can strongly affect PL efficiency.^9^ In our case, it is likely that Mn^2+^ ions fill the vacancies during crystal growth and thus reduce nonradiative decay channels. The overview HAADF‐STEM images clearly indicate that the NCs obtained with Mn^2+^ doping exhibit higher monodispesity as compared to undoped CsPbCl_3_ NCs (Figure 1 e,f). The undoped NCs appear to be polydisperse with a mixture of various morphologies including nanocubes and nanoplatelets of different lateral dimensions, whereas the Mn‐doped CsPbCl_3_ NCs are nearly monodisperse nanocubes with edge length of around 10–12 nm. This is consistent with the strong and sharp excitonic peak in Mn‐doped perovskite NCs. The excitonic peak becomes more prominent as the inhomogeneous broadening of doped NCs decreases. Moreover, the Stokes‐shift observed in ensemble spectra of doped NCs is smaller than that of undoped NCs, again related to the reduced inhomogeneous broadening (Figures 1 c,d). It is likely that the filling of vacancies by Mn^2+^ ions during the growth of NCs leads to isotropic particles; however, the change in the reaction conditions in the presence of a significant amount of Mn precursor cannot be excluded. Further proof for the reduced vacancies/traps in doped NCs was obtained by time‐resolved PL studies. The PL decay of the exciton stays the same in case of doped and undoped NCs (see Figure S2). This indicates that the increasing non‐radiative rate due to energy transfer balances with the decreasing non‐radiative rate due to trap filling. However, it should be noted that this holds only for the sub‐ensemble of emitting nanocrystals. The dark fraction of NCs might be actually large according to the low QY. Furthermore, the high‐resolution HAADF‐STEM images show that the undoped NCs are single crystalline (Figure 1 e inset). However in the case of doped NCs, although most of the particles are single crystalline at low doping concentration, we observe planar defects in some NCs (Figure 1 f inset and Figure S3).

To further understand the influence of Mn^2+^ doping on the optical properties as well as the crystallinity of host NCs, we prepared Mn^2+^‐doped CsPbCl_3_ NCs using different Mn/Pb precursor ratios (keeping the total concentration of the MnCl_2_‐PbCl_2_ mixture constant in all reactions). Note that similar results were obtained for Mn^2+^‐doped NCs prepared with a fixed amount of PbCl_2_ and a varying amount of MnCl_2_ (Figures S4 and S5). Figure 2 a shows a digital photograph of the colloidal dispersions of CsPbCl_3_ NCs as a function of increasing Mn to Pb feed ratio (0 to 23) under UV illumination. The colloidal dispersions exhibit different colors under UV excitation due to differences in the emission intensities of host excitons (blue) and dopants (orange). With increasing Mn^2+^ dopant concentration the emission becomes more orange due to efficient energy transfer from excitons to dopants. The color variation depending on the Mn dopant concentration is quantified by UV/Vis absorption and PL spectroscopy, as depicted in Figures 2 b,c, respectively (see Figure S4 for unnormalized data). The exciton peak in the absorption spectra becomes very prominent even at rather low doping concentration (Mn: Pb=0.5:1 to 1.7:1). However, the peak becomes broader with further increasing the Mn^2+^ dopant concentration, and at sufficiently high Mn^2+^ concentration (Mn: Pb=23:1, see Figure S6), no clear exciton absorption can be observed anymore. In addition, a gradual blue shift of the excitonic absorption as well as emission peaks can be clearly seen (Figures 2 c,d).

**Figure 2:**
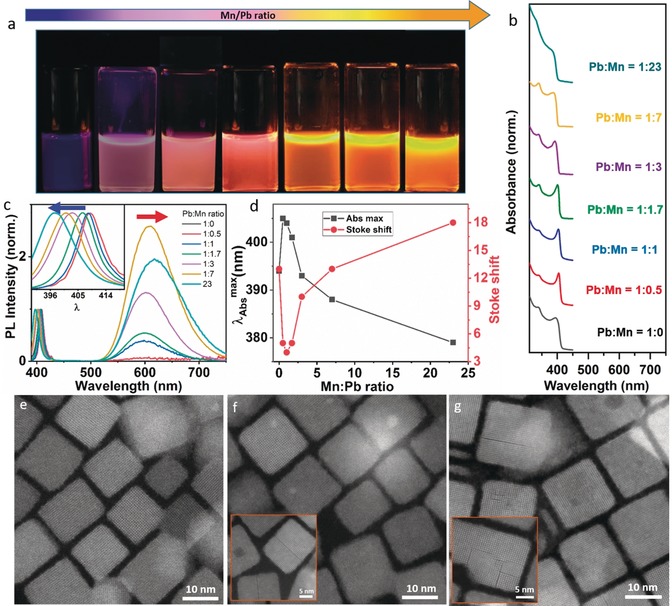
a) Digital photograph of colloidal dispersions of Mn doped CsPbCl_3_ NCs with increasing Mn to Pb feed ratio (0 to 23) under UV light. (b,c) corresponding absorption and PL spectra of the samples shown in (a). The inset of (c) shows the expansion of the excitonic PL which gradually blue shifts with increasing concentration of Mn dopant. d) Position of absorption maxima and Stokes shift as a function of increasing concentration of Mn dopant. e)–g) HAADF‐STEM image of Mn‐doped CsPbCl_3_ NCs obtained with Mn to Pb feed ratios 0.5:1, 1:1, and 1.7:1, respectively. The inset in (f) and (g) displays the corresponding high‐resolution HAADF‐STEM image, in which some particles show planar defects.

To understand these observations we investigated these samples of different Mn^2+^ dopant concentrations with high‐resolution HAADF‐STEM measurements (Figure 2 e–g and Figure S7). At very low Mn^2+^‐doping concentration (Mn: Pb=0.5:1), the particles are single crystalline nanocubes without defects similar to the undoped NCs (Figure 2 e). Moreover, the size distribution is improved in doped NCs. As a result, the exciton resonances are prominent at low dopant concentrations owing to reduced inhomogeneous broadening. At slightly increased dopant concentration (Mn: Pb=1:1), very few particles have single planar defects (Figure 2 f and Figure S8), otherwise most particles appear to be perfectly single crystalline. As the dopant concentration increases further (Mn: Pb=1.7:1–23:1), most particles show multiple planar defects as displayed in Figure 2 g, Figure 3 a,b (and Figure S7). From the atomically resolved HAADF‐STEM images, atomic dislocations are clear to see. Most likely these are identified as R.P. defect planes, in which (Pb/Mn)‐Cl atomic columns are shifted by half a unit cell at the border of the defect plane (Figure 3 c and Figure S3 b). Such R.P. defect planes were recently observed in mixed halide inorganic perovskite NCs arising from halide‐ion segregation caused by their size difference.^10^ In fact, these planar defects were also observed in pure CsPbBr_3_ NCs.^11^ In addition, theoretical studies suggest that these R.P. planar defects behave like a semiconductor–insulator–semiconductor junction.11a In the present case, it is likely that the formation of R.P. planes is triggered by the size difference between Mn^2+^ (1.6 Å) and Pb^2+^ (2.38 Å) arising from their lattice mismatch as well as the limited availability of Pb^2+^ for high Mn^2+^ concentrations. At the interface, the lattices are terminated with Cs and Cl atoms, which can be regarded as extra planes of CsCl inserted into the perovskite structure. These R.P. defect planes are different from conventional organic–inorganic R.P. 2D perovskites, in which long‐chain ligands act as spacers between stacked 2D perovskite sheets.^12^ The quantum confinement of carriers within the individual domains separated by R.P. defect planes in the host NCs is apparent from the blue shift of excitonic absorption and emission. Although the NCs appear as nanocubes, they can be considered as 3D stacked quantum‐confined NCs separated by R.P. defect planes.^10^ The excitons are confined to these subdomains separated by the R.P. planes in the host NCs. Such quantum confinement of the exciton in the subdomains also leads to an enhancement of the exciton oscillator strength which also contributes to the prominent exciton resonance in slightly doped NCs (Figure 2 b). The size of individual domains between R.P. defect planes decreases on average with increasing the dopant concentration (Figure S7). This increases the quantum confinement and leads to the observed gradual blue shift. However, the inhomogeneity of the individual domains at sufficiently high Mn^2+^ concentration causes a broadening of the excitonic absorption and emission. As a result, the Stokes‐shift of the exciton also increases with increasing dopant concentration (Figure 2 d). We rule out the possibility that the blue‐shift is caused by Mn^2+^‐induced lattice contraction because no significant variation in the average lattice spacing is observed (Figure S8). Furthermore, we carried out single‐particle PL measurements to understand the broad Mn d–d emission. We find that the single‐particle PL resembles the ensemble PL, meaning that the variations between individual NCs are negligible (Figure S9). However, the dopant PL peak position slightly varies depending on the Mn^2+^ concentration (Figure 2 c). Therefore, the broad Mn‐related emission originates from Mn^2+^ ions present at different sites (surface, near surface or core) of the crystal lattice as well as from Mn^2+^‐Mn^2+^ interactions.

**Figure 3:**
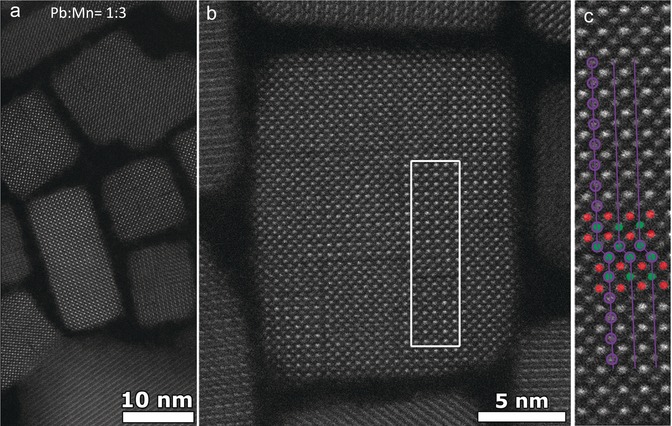
a) Overview HAADF‐STEM image of Mn‐doped CsPbCl_3_ NCs obtained with Mn to Pb feed ratio 3:1. b),c) corresponding atomically resolved HAADF‐STEM images showing R.P.‐defect planes (Pb/Mn‐Cl=red, Cs=green). The lattices are shifted half of the unit cell at the grain boundaries.

On the other hand, the doping concentration does not only influence the excitonic properties, but also the exciton‐to‐dopant energy transfer efficiency.6j As shown in Figure 2 c (and Figure S4), the intensity of Mn^2+^ orange emission increases initially with increasing dopant concentration because of improved energy transfer. However, excess doping leads to a decrease in the Mn^2+^ emission as a result of strong Mn^2+^–Mn^2+^ interactions in the lattice. We observe a maximum PLQY of 26 % for the doped colloidal NCs prepared using the precursors with Mn:Pb=3:1 (the content of Mn is approximately 3 % of Pb based on EDX analysis). Importantly, we find that the doped NCs are stable for several days (Figure S10). In addition, we have studied the exciton‐to‐dopant energy‐transfer efficiency as a function of optical band gap energy of the host perovskite NCs. For this, we added PbBr_2_ to the initial Mn doped CsPbCl_3_ matrix (Figure S11).6g According to the peak intensities of exciton and dopant emission, we found that the energy transfer was best in case of exciton emission at around 420 nm, likely a result of the strongest coupling with the Mn^2+^ dopant energy levels (Figure S11).

In summary, we have presented a systematic study on the influence of Mn‐doping on the excitonic properties of host perovskite NCs. We found that the slight doping of Mn^2+^ ions into perovskite NCs makes their excitonic absorption peak more prominent by improving the size dispersion of the NCs, and also enhances the PLQY of the excitonic emission by filling traps. We have shown that the doping of Mn^2+^ into perovskite NCs induces R.P. defect planes in host NCs. The number of R.P. defect planes in host NCs increases with increasing the dopant concentration and thus the size of the individual domains separated by these defect planes is reduced. Additionally, an increasing amount of Mn doping leads to a blue shift and broadening of the excitonic absorption and emission because of R.P. defect‐induced quantum confinement within the NCs. This work not only provides insights into the effect of Mn^2+^‐doping on the excitonic properties of host perovskites, but also opens door for the one‐pot synthesis of doped perovskite NCs by ultrasonication.

## Conflict of interest

The authors declare no conflict of interest.

## Supporting information

As a service to our authors and readers, this journal provides supporting information supplied by the authors. Such materials are peer reviewed and may be re‐organized for online delivery, but are not copy‐edited or typeset. Technical support issues arising from supporting information (other than missing files) should be addressed to the authors.

Supplementary
