# Supplementary material for: Manganese‐Doping‐Induced Quantum Confinement within Host Perovskite Nanocrystals through Ruddlesden–Popper Defects
Source: Angew Chem Int Ed Engl. 2020 Mar 5;59(17):6794–9. doi: 10.1002/anie.201914473 (PMC7186832; doi:10.1002/anie.201914473)
Supplement: Supplementary file 1 — Supplementary [file ANIE-59-6794-s001.pdf]

Angewandte Chemie
Eine Zeitschrift der Gesellschaft Deutscher Chemiker

Supporting Information

Manganese-Doping-Induced Quantum Confinement within Host Perovskite Nanocrystals through Ruddlesden–Popper Defects

Sharmistha Paul, Eva Bladt, Alexander F. Richter, Markus Döblinger, Yu Tong, He Huang, Amrita Dey, Sara Bals, Tushar Debnath, Lakshminarayana Polavarapu,* and Jochen Feldmann*

anie_201914473_sm_miscellaneous_information.pdf

Supporting Information

1. Materials and methods:

CsOAc (Cesium acetate, 99%), $PbCl_2$ (Lead(II) chloride, 98%), $PbBr_2$ (Lead(II) bromide, 98%), 1-octadecene (technical grade 90%), $MnCl_2$ (Manganese(II) chloride, 99%), oleylamine (technical grade 70%), oleic acid (technical grade 90%) and hexane (HPLC, grade ≥97.0%, GC) were purchased from Sigma-Aldrich. All chemicals were used as received.

1.1. Synthesis:

(a) Synthesis of $CsPbCl_3$ nanocubes:

In a typical colloidal synthesis of $CsPbCl_3$, 0.75 ml of oleylamine, 0.75 ml of oleic acid and 10 ml of 1-octadecene were added to a mixture of 0.06 mmol CsOAc and 0.24 mmol $PbCl_2$ precursor powders and then processed by tip-sonication (SONOPULS HD 3100, BANDELIN) at a power of 30 W for 15 minutes. During the reaction, the colorless reaction medium gradually transformed into a turbid solution, which exhibits a weak fluorescence under UV-light excitation. After 15 minutes, the reaction medium was cooled down with ice-cold water and the unreacted precursors and excess ligands were removed by centrifugation at a speed of 10000 rpm for 10 min and then the particles were re-dispersed in 2 ml of hexane. The obtained colloidal solution contained a small portion of nanocubes together with some big particles. To separate the expected nanocubes from the big particles, the colloidal solution was centrifuged at a speed of 6500 rpm for 5 min and then the supernatant was collected for further purification. 1 ml of acetone was added to the supernatant and then centrifuged at 3000 rpm for 5 minutes to precipitate the nanoparticles. The residue was dissolved in 1 ml of hexane and stored for further experiments. This purification process remained the same for all of the other synthesis of nanocrystals. Furthermore, it was found that the nanocubes yield is higher when the ultrasonication was carried out by placing the glass bottle on a metal stand rather than an insulating stand.

(b) Synthesis of Mn-doped $CsPbCl_3$ nanocubes:

In a typical colloidal synthesis, 0.75 ml of oleylamine, 0.75 ml of oleic acid and 10 ml of 1-octadecene were added to a constant amount of 0.06 mmol CsOAc and $PbCl_2$ - $MnCl_2$ precursor powder by varying the Pb to Mn ratio (keeping the total amount of 0.24 mmol as constant) and then processed by tip-sonication as described above. During the sonication process, the colorless reaction medium gradually transformed into a yellowish and then a turbid solution. Under UV light, the turbid solution emits orange color, which indicates the formation of Mn-doped perovskite colloidal crystals. After the completion of the reaction, same purification steps were followed as described for the $CsPbCl_3$ nanocubes.

The concentration of $MnCl_2$ precursor used during the reaction was varied in two ways. First, the amount of CsOAc precursor was kept constant, while the ratio of $PbCl_2$ to $MnCl_2$ precursors was

changed (type 1). Second, the amount of CsOAc and $PbCl_2$ was kept constant, while the $MnCl_2$ concentration was varied (type 2). For each case, the used amount of ligands and solvents was same as mentioned above. The used concentrations for the two variants are listed the following table and the results were identical.

Table S1: Amount of precursors (in milimole) taken for the synthesis of Mn-doped $CsPbCl_3$ perovskites for type 1 and type 2 approach.

Type 1		
CsOAc (mmol)	$PbCl_2$ (mmol)	$MnCl_2$ (mmol)
0.06	0.16	0.08
0.06	0.12	0.12
0.06	0.09	0.15
0.06	0.06	0.18
0.06	0.03	0.21
0.06	0.01	0.23

Type 2		
CsOAc (mmol)	$PbCl_2$ (mmol)	$MnCl_2$ (mmol)
0.06	0.09	0.10
0.06	0.09	0.15
0.06	0.09	0.30
0.06	0.09	0.60

(c) Preparation of $PbBr_2$ solution:

A yellowish $PbBr_2$ solution was prepared by dissolving 4 mmol of PbBr2 precursor powder in a mixture of 100 ml of hexane, 4 ml of oleylamine and 4 ml of oleic acid at 120 °C under continuous stirring with 750 rpm for 2 hours. After complete dissolution of the precursor powder, the solution was kept for further use.

(d) Composition tuning through a halide ion exchange reaction with $PbBr_2$ solution addition:

At this point, a particular amount of $PbBr_2$ solution was added to the parent Mn-doped $CsPbCl_3$ nanocube dispersion to initiate a halide ion exchange and the sample container was shaken after each addition. The measurements of absorption and photoluminescence intensity were taken after each addition. The amount of the halide source was varied from 3 µl to 18 µl to tune the composition of the cubes and the resulting photoluminescence.

1.2. Optimization Process:

(a) Variation of precursors and ligand: From the optimization process, it was observed that the Cs to Pb ratio has direct impact on the optical property of the Mn-doped $CsPbCl_3$ nanocrystals. Keeping the amount of $MnCl_2$ precursor and solvent constant, the ratio of Cs to Pb precursor was varied along with the volume of ligands used. During this optimization process, it was observed that the reaction required the presence of both oleylamine and oleic acid as ligands. There was a drastic impact on the PLQY with the variation of the ratio of Cs to Pb and also with the ligand. Details of the experimental results are summarized in Table S2.

Table S2. Summary of the results obtained under different reaction conditions for the synthesis of Mn-doped $CsPbCl_3$ nanocrystal for a particular Mn concentration (0.18mmol).

System	Cesium precursor (mmol)	Lead precursor (mmol)	Oleylamine (ml)	Oleic acid (ml)	PLQY (in %)
Mn-doped $CsPbCl_3$	0.04	0.06	0.40	0.40	10
	0.04	0.06	0.50	0.50	20
	0.04	0.06	1.00	1.00	17
	0.06	0.06	1.00	1.00	25
	0.06	0.06	1.00	0	Fail
	0.06	0.06	0.75	0.75	31
	0.10	0.06	0.75	0.75	8.48

(b) Variation of the time and power of the tip-sonicator: The power of the SONOPULS HD 3100 ultrasonicator was also varied to obtain the highest PLQY along with the reaction time. During this process, it was observed that the emission from the Mn-doped systems appears after 10 minutes and the reaction medium became saturated within 15 minutes. If the reaction time was increased further, the performance of the nanocrystals did not improve. At high power, the reaction medium became more blackish and there was no formation of nanocrystals.

2. Characterization:

(a) UV- Vis absorption and Photoluminescence (PL) Spectra:

The UV-Vis absorption of $CsPbCl_3$ and Mn-doped $CsPbCl_3$ perovskite nanocubes and nanoplatelets dispersions was measured by a Cary 5000 UV-Vis-NIR spectrophotometer and the photoluminescence spectra were collected with a Varian Cary Eclipse fluorescence spectrophotometer (Agilent Technologies). The photoluminescence quantum yields (PLQYs) were measured using an integrating sphere embedded in a Fluorolog-3 FL3-22 spectrometer (Horiba Jobin Yvon GmbH). For absorbance, PL and PLQY measurement, purified colloidal samples dispersed in 3 ml hexane were filled in a cuvette of 1 cm path length.

(b) Transmission electron microscopy (TEM):

Overview and high resolution HAADF-STEM images were acquired using a cubed FEI Titan microscope operating at 300 kV (at Department of Chemistry, LMU Munich). Specimens for the TEM imaging were prepared on a carbon coated copper grid by dropping 10 μL of dilute colloidal dispersions of each sample. For elemental analysis with this instrument, energy-dispersive X-ray (EDX) analysis was performed with a Si(Li) detector. Atomically resolved HAADF-STEM images (inset of figure 1e and figure 3) were acquired at EMAT - Electron microscopy for Materials science, University of Antwerp, using a cubed FEI Titan microscope operating at 300 kV, using a probe semiconvergence angle of ~21 mrad (obtained one week after sample preparation). To obtain atomically resolved images, the samples were purified by reprecipitation in acetone.

(c) Single particle photoluminescence measurements:

The PL of single NCs was detected using a self-built micro-PL setup. The NC dispersion was diluted and spin-coated onto a silicon substrate in a dynamic way ($5000\ rpm$, $50\ \mu l$). The sample is excited under ambient conditions via an objective (Olympus, $40x$, NA 0.55) using a laser at $380\ nm$ (SuperK Extreme, NKT Photonics). Emission is detected via the same objective and directed onto an imaging spectrograph (Acton SpectraPro SP-2300 with a Pixis 400 CCD camera, Princeton Instruments). The reflecting laser beam is blocked using dichroic and long pass filters.

(d) Streak camera measurements:

The PL decay was measured using a Hamamatsu streak camera system (C10910-05). The sample was excited with a 385 nm fs-laser pulse, which is the second harmonic of a Ti:Sapphire laser (Mira Optima 900-F, Coherent).

3. Results and Discussions:

3.1. Single-molecule spectroscopy

We performed single particle spectroscopy to unravel if the local lattice environment of the Mn dopant varies between individual NCs. Figure S9a shows an image of single NCs and figure S9b

compares the Mn-related PL of several single particles with the ensemble emission of Mn-$CsPbCl_3$ NCs in solution. It can be seen that the PL spectra of all particles are quite similar to the ensemble PL of the Mn-doped NCs, in terms of both peak position as well as bandwidth (FWHM). However, it is well known in the literature that the PL peak positions and bandwidths of the single particle luminescence of the commonly studied Mn doped II-VI NCs are sensitive to local lattice environment. This sensitivity normally results in a wide tunability of the single particle luminescence from the green to red region of the spectrum, with significant spectral narrowing. This observation has been explained with the presence of a large number of inequivalent sites for the dopant (from core to the surface of the Mn doped NCs) leading to the variation of the ligand field transition (i.e. different $^4T_1$-$^6A_1$ energy gap). Therefore, we expected varying single particle luminescence spectra of the Mn doped perovskites. The absence of any significant spectral tunability as well as a comparable FWHM suggests that in contrast to II-VI NCs Mn doping in perovskite NCs produces relatively similar local lattice environments for the dopant at a particular doping concentration. This observation indicates that the ligand-assisted ultrasonication procedure employed in the present work for doped perovskite NCs synthesis, yields uniform lattice environments for the dopant within the perovskite NCs.

3.2. Tuning of energy transfer efficiency through halide ion exchange

To study the exciton to Mn energy transfer (ET) process as a function of bandgap of the perovskite NCs, we added different concentration of $PbBr_2$ to Mn-doped $CsPbCl_3$ NCs, thus producing mixed halide Mn-doped perovskite NCs. We investigated optical properties by monitoring the PL spectra (figure S11a). It is evident from the PL spectra that with increasing $PbBr_2$ concentration, the exciton emission red shifts due to the incorporation of Br into the perovskite crystal, which lowers the bandgap. Furthermore, with increasing Br-content, a small but clear red-shift of the Mn emission peak has been observed, along with spectral broadening of Mn-related PL (figure S11b). This observation indicates that the local environment of the Mn dopant is sensitive to the Br-content in the lattice which results in an alternation of the ligand-field splitting of Mn as well as in the introduction of some uncertainty in the Mn energy levels, which leads to an increased FWHM. To unravel the energy transfer efficiency between excitonic and Mn energy levels upon increased $PbBr_2$ concentration, the relative peak intensity ratio of Mn PL and excitonic PL has been computed (Figure S11a inset). Initially, the addition of $PbBr_2$ until a certain concentration results in increased relative Mn PL intensity, which can be attributed likely due to strongest coupling between excitonic and Mn energy levels and therefore, the exciton to Mn energy transfer becomes more efficient. Further addition of $PbBr_2$ results in a reduction of the energy transfer efficiency owing to an increased back energy transfer (BET) process as the excitonic and Mn energy levels comes close enough. Figure S11c schematically depicts such energy transfer and back energy transfer processes for increasing Br content in the Mn-doped perovskite NCs.

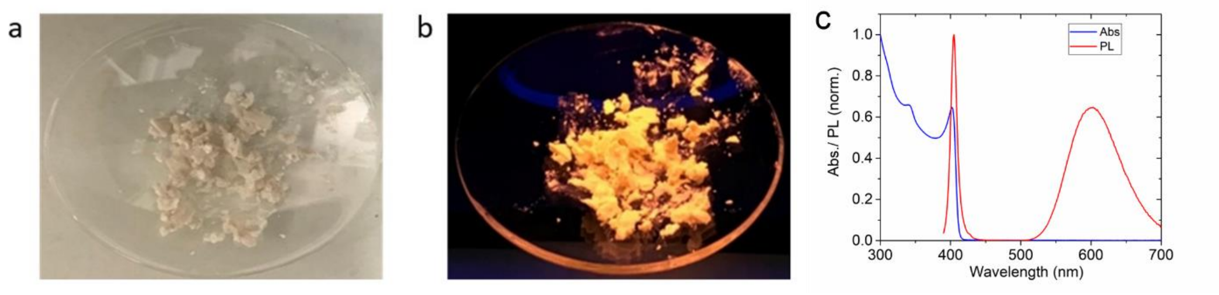

Figure S1: Large scale synthetic product of Mn-doped $CsPbCl_3$ nanocubes in presence of a) normal light and b) UV light. c) Absorption and PL spectra of the corresponding NCs. The precursors for the reaction were scaled up 50 times and tip-sonicated for an hour. The normal purification method was followed to get the pure nanocubes and dried in presence of air.

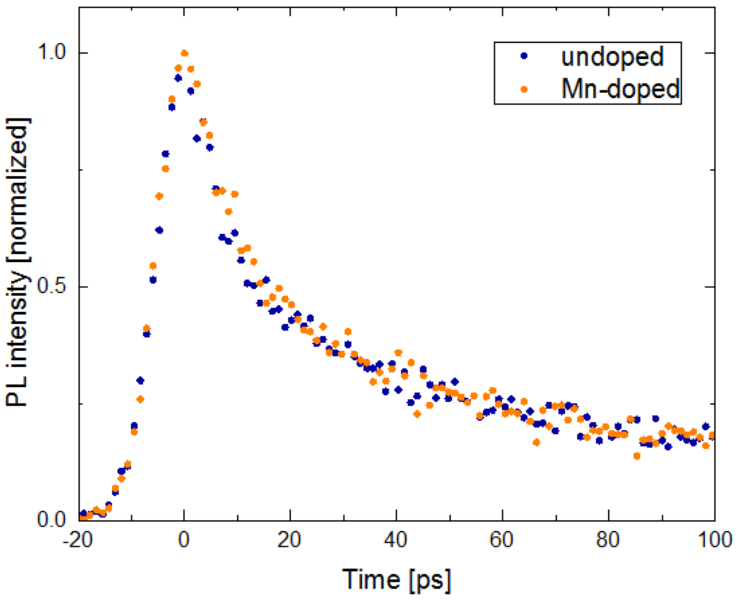

Figure S2: Time-resolved excitonic PL (at 400 nm) decay traces of undoped and Mn-doped (Mn:Pb= 3:1) $CsPbCl_3$ NCs after excitation with a 380 nm laser pulse.

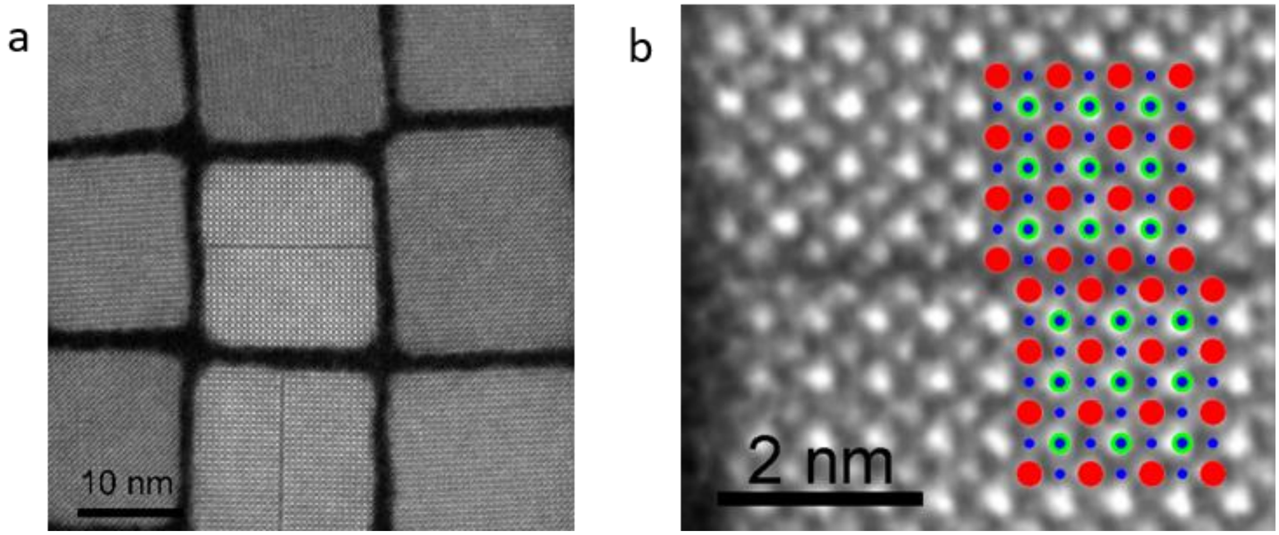

Figure S3: a) HAADF- STEM image of Mn-doped $CsPbCl_3$ NCs obtained with Mn to Pb feed ratio 1.7:1, showing single line defect along the crystal. B) Atomically resolved HAADF- STEM images of Mn-doped $CsPbCl_3$ NCs (Mn:Pb= 1:1.7) showing Ruddlesden popper defect planes Pb/Mn=green, Cs=red, Cl=blue). The lattices are shifted half of the unit cell at the grain boundaries.

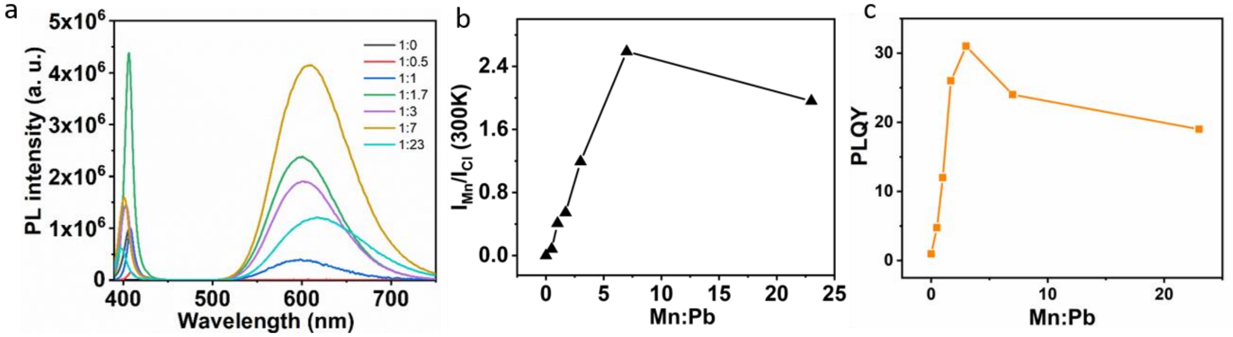

Figure S4: a) Unnormalized PL spectra of successfully prepared Mn-doped $CsPbCl_3$ nanocubes using different ratio of Pb to Mn precursor powder. b) Ratio of Mn PL intensity to excition PL intensity as a function of Mn concentration. c) Total PLQY as a function of Mn concentration which has a maxima at 1:3 Pb to Mn doping.

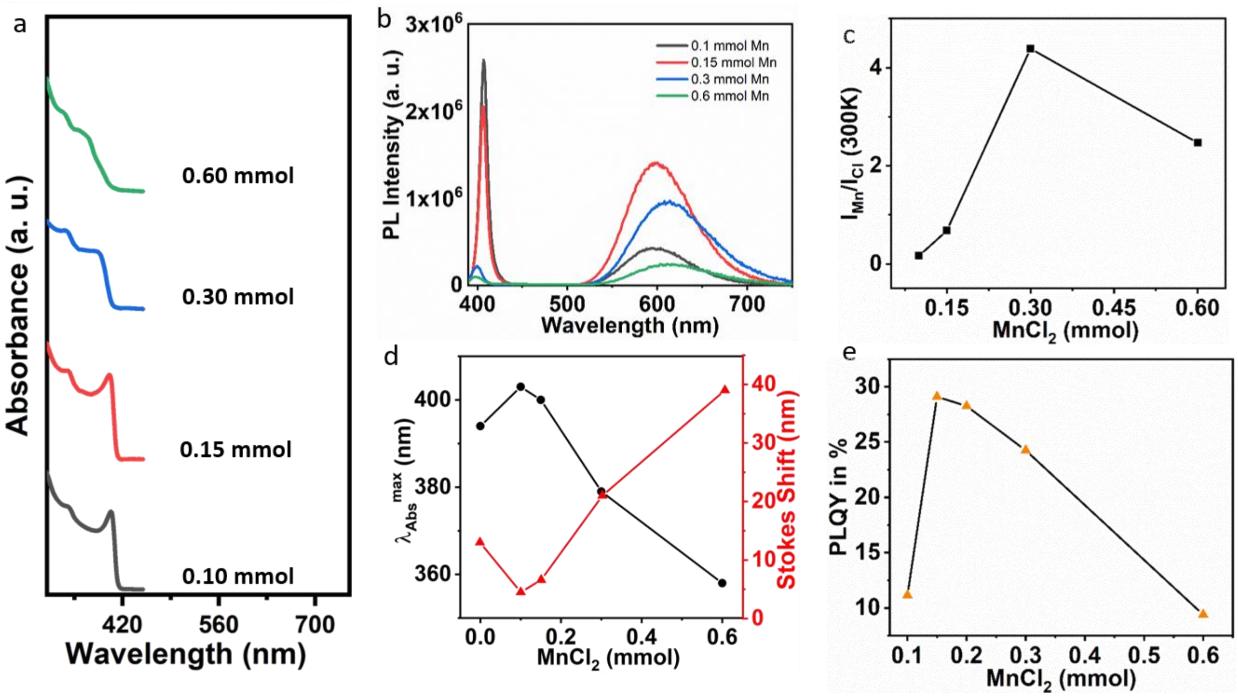

Figure S5: a, b) Optical absorption and PL spectra of colloidal dispersions of Mn doped $CsPbCl_3$ NCs with increasing Mn concentration (0.1 to 0.6 mmol) keeping same Pb concentration. c) Ratio of Mn PL intensity to excition PL intensity as a function of Mn concentration. d) Position of absorption maxima and Stokes shift as a function of increasing concentration of Mn dopant, which clearly shows blue-shift in the absorption spectra with Mn concentration due to Ruddlesden popper (RP) defect induced quantum confined effect. The Stokes shift is also increases with Mn concentration due to inhomogeneous broadening at high Mn concentration. e) Total PLQY as a function of Mn concentration (at fixed Pb concentration) which has maxima at 0.15 mmol Mn doping.

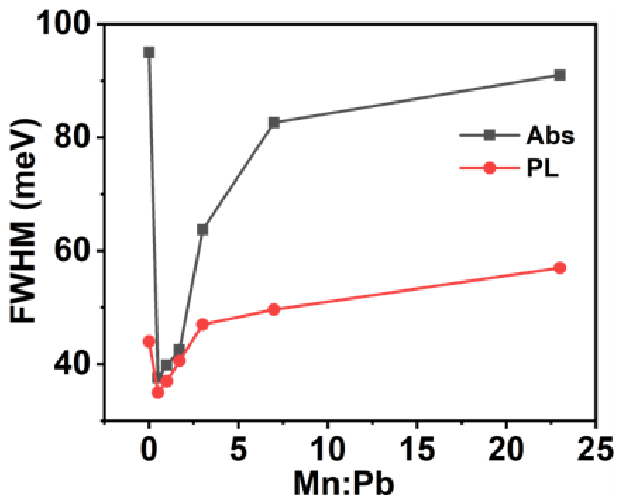

Figure S6: FWHM of both absorption (1st exciton peak) and PL spectra as a function of Mn concentration with increasing Mn:Pb ratio.

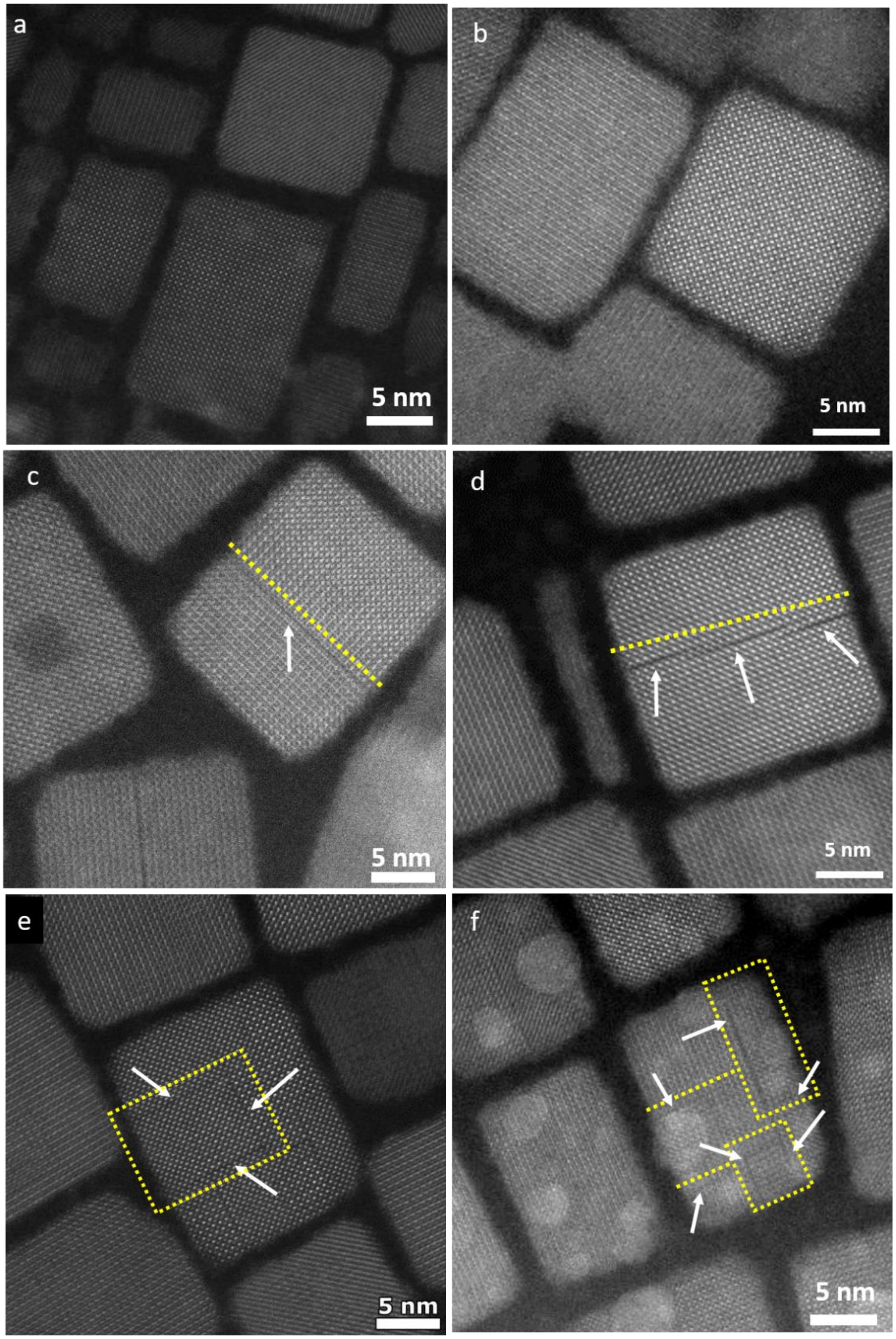

Figure S7: HAADF- STEM image of Mn-doped $CsPbCl_3$ NCs obtained with Mn to Pb feed ratio a) 0, b) 0.5, c) 1, d) 1.7, e) 3 and f) 23. White arrows indicate the RP induced line defects while the yellow dotted box indicates the quantum confined domains within the host NCs. Scale bar 5 nm. These images were acquired after purification of samples by reprecipitation in acetone. The size of the particles for all Mn-doped $CsPbCl_3$ NCs are ~10-15 nm.

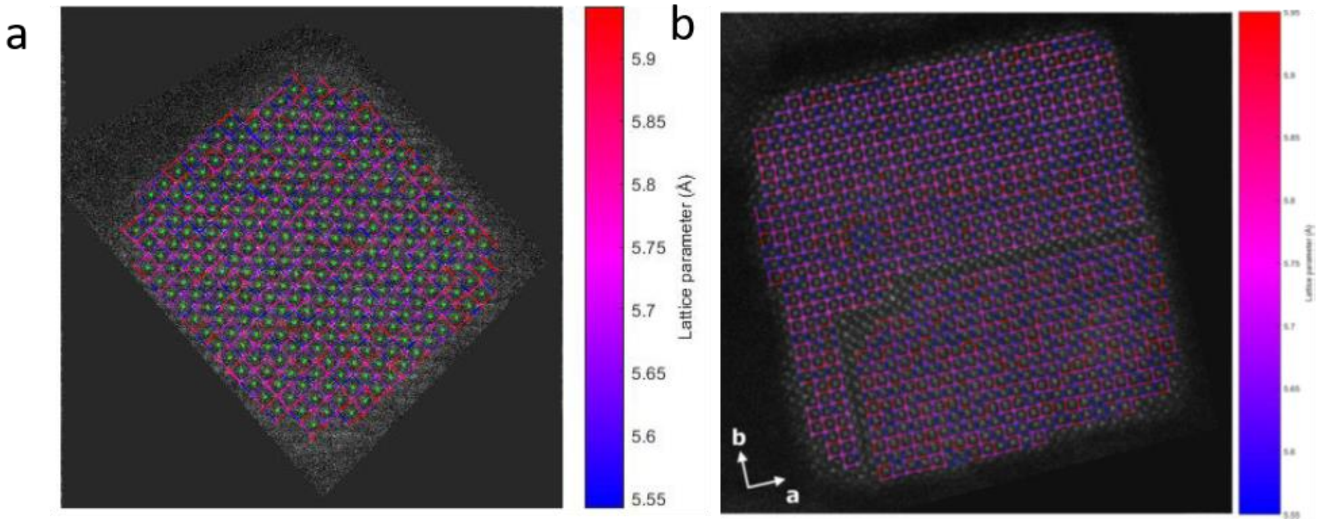

Figure S8: Interactomic distances between the Pb/halide atomic columns in **a)** undoped with |a| = (5.74 ± 0.05) Å and |b| = (5.65 ± 0.05) Å **b)** Mn doped $CsPbCl_3$ NCs (Mn to Pb feed ratios 3:1) with average distances above the planar defect are |a| = (5.75 ± 0.05) Å and |b| = (5.58 ± 0.05) Å; and the average distances below the planar defect are |a| = (5.76 ± 0.05) Å and |b| = (5.67 ± 0.05) Å.

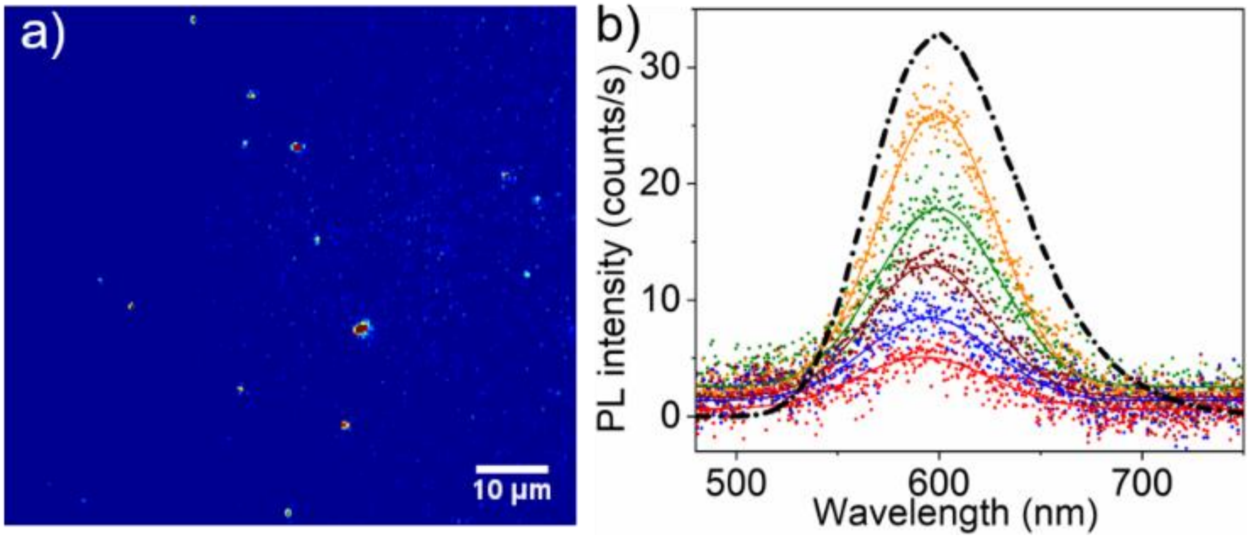

Figure S9: a) Single particle PL image and b) spectra of several single particles showing the Mn-related luminescence (dots correspond to raw data and solid lines to a Gaussian fit). The spectrum of the ensemble emission in solution is also shown for comparison (black dot-dashed line)

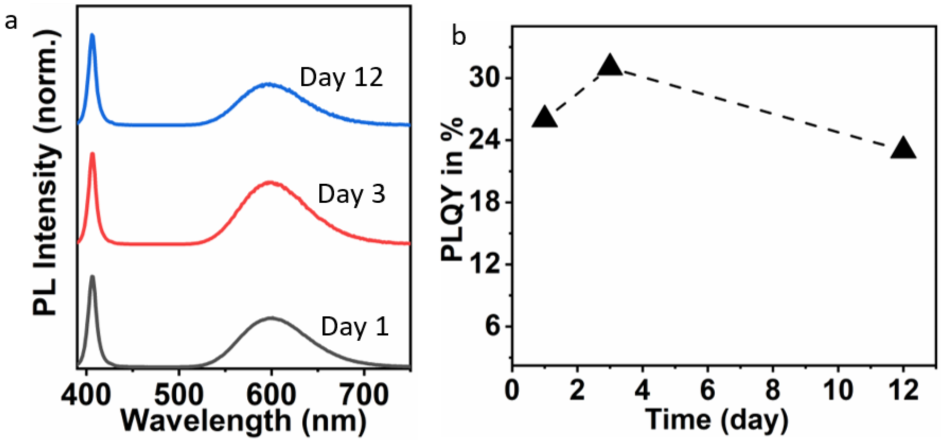

Figure S10: Long-term stability of successfully prepared Mn-doped $CsPbCl_3$ nanocubes dispersed in hexane. a) PL spectra of one particular Mn-doped $CsPbCl_3$ nanocubes taken at three different days. b) Corresponding PLQY.

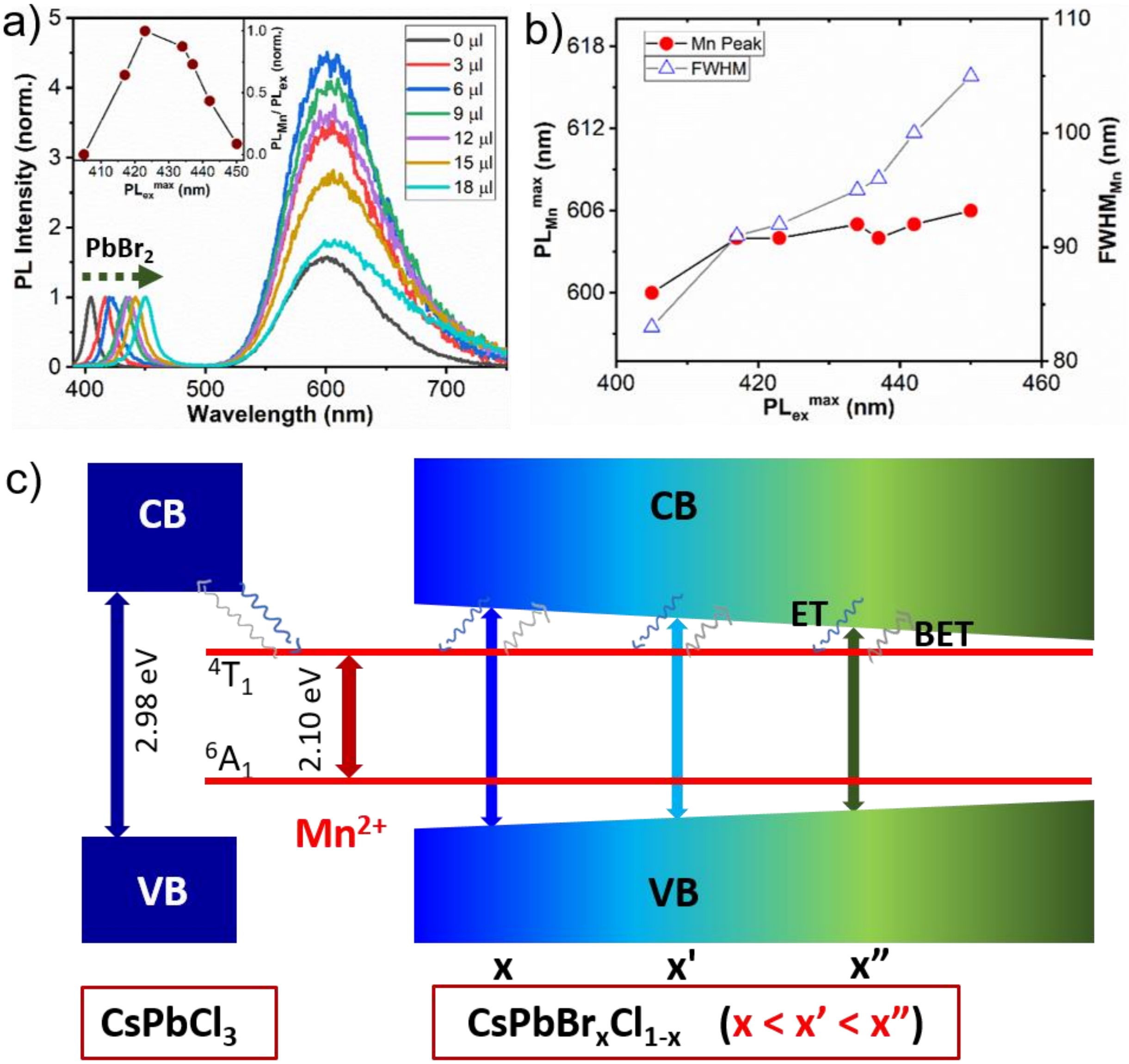

Figure S11: Addition of $PbBr_2$ to Mn-doped $CsPbCl_3$ NCs to study the better coupling between excitonic and Mn energy levels for efficient energy transfer (ET). (a) PL spectra of Mn-doped $CsPbCl_3$ NCs with increasing concentration of $PbBr_2$ solution (0.04 M). (Inset) Relative peak intensity ratio of Mn PL and excitonic PL as a function of excitonic PL peak wavelength. (b) Change in Mn PL peak position and full-width at half maxima (FWHM) position of Mn PL as a function of excitonic PL position. (c) Schematic illustration of ET and back energy transfer (BET) process between excitonic and Mn energy levels upon decrement of the perovskite NCs bandgap due to increased Br-content.
